# Detection of viral antibodies in camel sera using magnetic particle spectroscopy

**DOI:** 10.1007/s00253-023-12513-4

**Published:** 2023-04-15

**Authors:** Bernhard Friedrich, Patrick Vogel, Martin A. Rückert, Stefan Lyer, Johanna Günther, Ulrich Wernery, Sunitha Joseph, Judith Müller, Volker C. Behr, Christoph Alexiou, Rainer Tietze

**Affiliations:** 1 Department of Otorhinolaryngology, Head and Neck Surgery, Section of Experimental Oncology and Nanomedicine (SEON), Else Kröner-Fresenius-Stiftung Professorship, Universitätsklinikum Erlangen, Erlangen, Germany; 2 Department of Experimental Physics 5 (Biophysics), Julius-Maximilians-University Würzburg, Würzburg, Germany; 3 Department of Otorhinolaryngology, Head and Neck Surgery, Section of Experimental Oncology and Nanomedicine (SEON), Professorship for AI-Controlled Nanomaterials, University Hospital Erlangen, Erlangen, Germany; 4 Central Veterinary Research Laboratory, P.O. Box 597, Dubai, UAE; 5 Generatio - Center for Animal Genetics, Heidelberg, Germany

**Keywords:** SARS-Cov-2, MNPs, Camels, MPS, COMPASS, Antibody detection, Rapid bioassay

## Abstract

**Abstract:**

Pandemics like SARS-Cov-2 very frequently have their origin in different animals and in particular herds of camels could be a source of zoonotic diseases. This study took advantage on a highly sensitive and adaptable method for the fast and reliable detection of viral antibodies in camels using low-cost equipment. Magnetic nanoparticles (MNP) have high variability in their functionalization with different peptides and proteins. We confirm that 3-aminopropyl triethoxysilane (APTES)-coated MNP could be functionalized with viral proteins. The protein loading could be confirmed by simple loading controls using FACS-analysis (*p* < 0.05). Complementary combination of antigen and antibody yields in a significant signal increase could be proven by both FACS and COMPASS. However, COMPASS needs only a few seconds for the measurement. In COMPASS, the phase *φ*_n_ on selected critical point of the fifth higher harmonic (*n* = 5th). Here, positive sera display highly significant signal increase over the control or negative sera. Furthermore, a clear distinction could be made in antibody detection as an immune response to closely related viruses (SARS-CoV2 and MERS). Using modified MNPs along with COMPASS offers a fast and reliable method that is less cost intensive than current technologies and offers the possibility to be quickly adapted in case of new occurring viral infections.

**Key points:**

*• COMPASS (critical offset magnetic particle spectroscopy) allows the fast detection of antibodies.*

*• Magnetic nanoparticles can be adapted by exchange of the linked bait molecule.*

*• Antibodies could be detected in camel sera without washing steps within seconds.*

## Introduction

The dromedary or “one-humped camel” is a versatile livestock animal that is kept mainly in Near and Middle East, different parts of Africa and some Asian regions (Faye 2020). The reason for the special immunological resistance of these animals can be traced back to components of their blood. Special antibodies of camels, which are the basis for the so-called nanobodies, differ from those of other mammals, because they lack the light chains in the structure, they are smaller and more stable, and penetrate deep into infected tissue (Al-Numair et al. 2022). Camel antibodies cause a strong immunogenic response and neutralize a wide range of viral antigens effectively (Kandeel and Al-Mubarak 2022). The blood serum of camels is also used in human medicine, for example, to obtain highly effective antidotes from it (El-Fakharany et al. 2012). However, some aspects of the immunological disposition of the dromedary also pose a risk to human health. Thus, the animals are a favorable intermediate host for some viruses, which makes dromedary husbandry a relevant risk factor for zoonotic disease transmission (Zhu et al. 2019). In the last decades, some significant zoonotic diseases have been caused by corona viruses (Fung et al. 2021) and camels are reservoirs and carriers of various coronavirus strains (Kandeel and Al-Mubarak 2022). During the outbreak of MERS (Middle East Respiratory Syndrome) in 2012, the dromedary was identified as the intermediate host from which the spillover of the virus to humans originated (Zhu et al. 2019). On the other hand, prolonged droughts and unfavorable forage conditions increase the need for camels as an enduring farm animal in the so affected regions worldwide. Hence, the growing trade of camels and camel products further enhances the risk of virus transmission to humans and other animals. Due to the lack of effective vaccines against relevant viral diseases for camels, early detection of antibodies is an important parameter to prevent the spread of zoonotic epidemics (Kandeel and Al-Mubarak 2022). Currently, screening for antibodies against certain infections, especially viral infections in large animal and human populations, is done in laboratories using immunochemical methods that are expensive and, above all, time-consuming. Together with the need for transportation and probe logistics, this sums up to unnecessarily delaying the need for acute action.

For efficient onsite screening, there is a lack of portable devices that can be used directly on site for early detection (Vidic et al. 2017). The need of robust and fast detection has been addressed by a number of new approaches in biosensors (Tewari et al. 2021).

The work presented here shows a rapid testing of camel serum for coronavirus antibodies using a very recently developed modified magnetic particle spectroscopy (MPS) (Biederer et al. 2009) method utilizing the high sensitivity of offset field induced critical points (Critical Offset Magnetic PArticle SpectroScopy – COMPASS) (Vogel et al. 2022). This novel method not only provides a robust and fast sample analysis with a portable and easy-to-use device but also shows sensitivities competitive with gold standard measurement methods such as ELISA. Magnetic nanoparticles (MNPs) have shown to be a very versatile tool for diagnostic and therapeutic purposes in different medical fields (Baumann et al. 2005; Friedrich et al. 2022a; Friedrich et al. 2022b; Kappes et al. 2022; Kluchova et al. 2009; Mühlberger et al. 2019; Wei et al. 2015). COMPASS uses the magnetization response of functionalized MNP ensembles on time-varying (AC magnetic fields) and specific offset magnetic fields (DC or offset magnetic fields) to determine specific information about MNP mobility in their environment and thus the conjugations of chemical or biological compounds on their surface. This behavior fundamentally changes when the particles, previously provided with antigens as bait proteins, encounter complementary binding antibodies in the serum sample to be tested. However, due to this novel measurement method, minimal size changes of the MNP resulting from antigen–antibody binding at the particle surface can be detected with a high sensitivity and robustness within seconds offering rapid and mobile testing devices. With minor hardware adjustments, the detection limit shifts even below the performance of classical immunological methods such as ELISA. The simple design and the robust sample handling make an application in the veterinary epidemiological field very attractive.

In our study, sera of differently immunized camels were directly measured in the COMPASS device after less than 1 min of incubation with functionalized MNPs. The deployed particles exposing different antigens were used in the measurement procedure to determine to what extent selective binding can be detected and to what extent the presence of virus-specific antibodies is evident with high selectivity, knowing that, in contrast to other immunological determination procedures, no purification steps are used in this measurement procedure.

## Materials and method

### Materials

Iron(II) chloride tetrahydrate, iron(III) chloride hexahydrate, and bovine serum albumin lyophilized powder were purchased from Merck KGaA, Germany. Ammonia, 25% (v/v) water solution, (3-aminoprpyl)triethoxysilane (APTES), hydrochloric acid concentrated, nitric acid, sodium hydroxide, hydrochloric acid 1N, and boric acid were purchased from Carl Roth GmbH + Co, KG, Germany. N-Succinimidyl bromoacetate was supplied by VWR International GmbH, Germany. Viral proteins and antibodies were purchased from Sino Biological, China. Deionized water was produced using a Merck Milli-Q purification system. All reagents were used without further purification.

### Immunization of camels

**Scheme 1:**
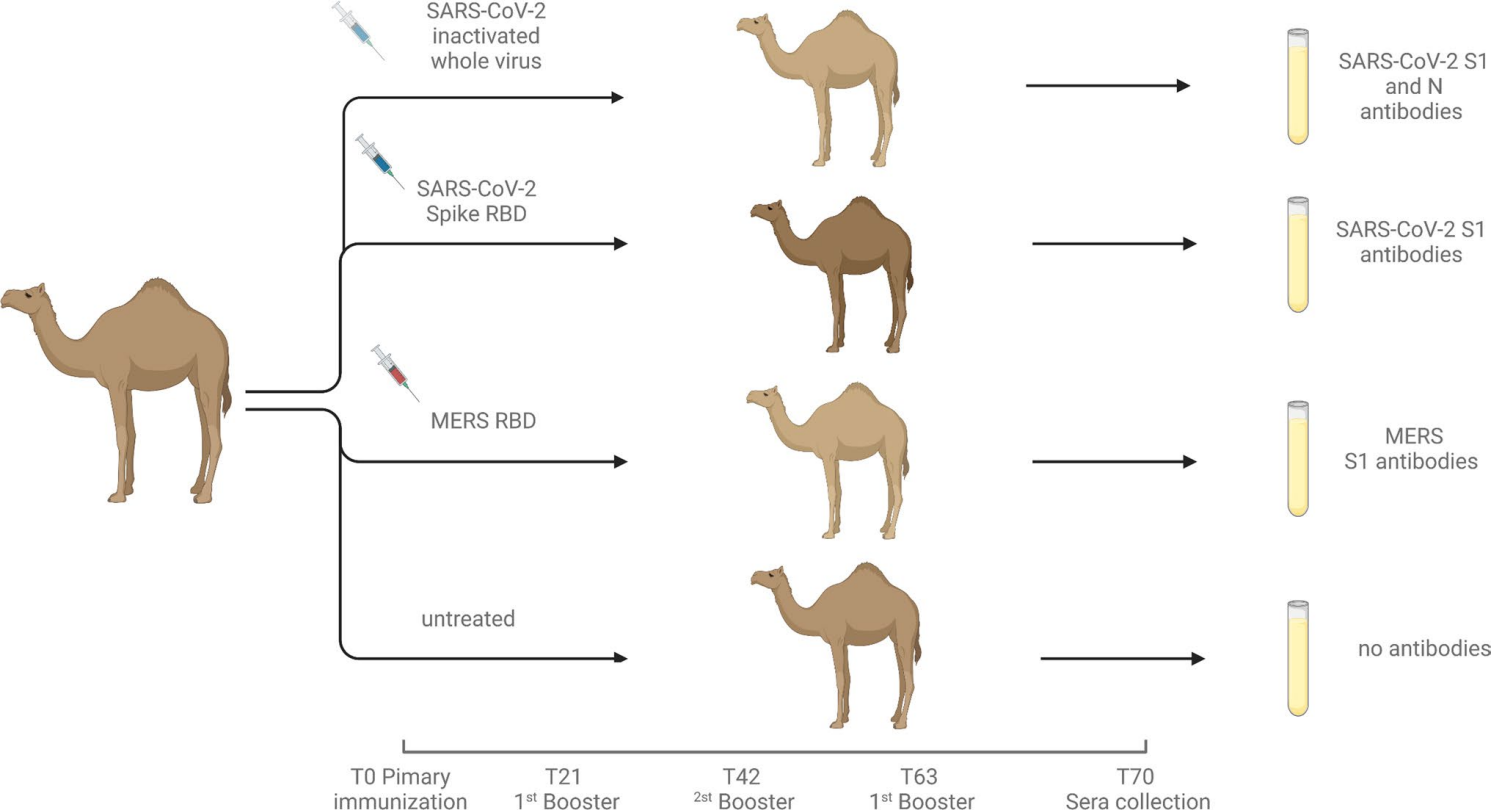
Immunization scheme of the deployed animals. Created with BioRender.com

Twenty-five dromedary camels of different gender and different age are kept at the Central Veterinary Research Laboratory (CVRL), Dubai, UAE, for research purposes. They are housed in 7 different shaded pens and are fed with alfalfa hay at libitum and 1.0-kg dry pellet per day. They have access to automatic water drinkers. The animals are healthy and there is no other camel herd existing in the vicinity of 30 km, and therefore, no potential threat of zoonotic diseases was obvious. The health of the dromedary camel herd is regularly monitored by 2 veterinarians. In total, 8 dromedary camels (*Camelus dromedarius*) kept at CVRL were selected to raise camel immune sera against SARS-Cov-2 and MERS. The camels were split randomly into 4 groups comprising 2 camels for each antigen and 2 camels as controls (Scheme 1). Each group was designated to receive 0.2 mg SARS-Cov-2 S1-RBD, 0.2 mg MERS-Cov S1-RBD, inactivated SARS-Cov-2 whole virus (10^6.25^ TCID_50_/ml), and Imject™ Alum without an immunogen (control group) respectively. Seven immunizations were performed subcutaneously for all groups except the controls, at a 2-week interval each containing 1.0 ml of antigen mixed in Imject™ Alum, Thermo Scientific, USA. Before and after each immunization blood was withdrawn from the jugular vein of each camel, the blood was allowed to clot at room temperature and sera were collected and stored at – 20 °C.

### Determination of antibodies after immunization

The strength of the immune response was determined using the cPass™ SARS-Cov-2 Neutralization Antibody Detection Kit, GenScript, China, and anti-MERS-Cov ELISA (IgG), Euroimmune, Germany. The test was performed in accordance with the instructions in the kit.

### Synthesis and functionalization of MNP-antigen

**Fig. 1:**
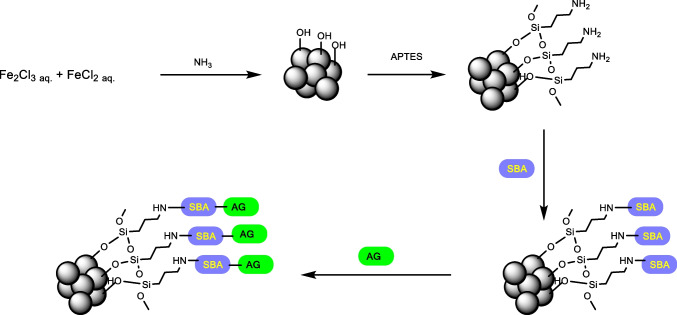
Reaction scheme of the particle functionalization. Starting alkaline precipitation the core–shell coating with 3-aminopropyl triethoxysilane (APTES) occurs, following the activation by the thiolreactive N-succinimidyl bromoacetate (SBA) and the final linking of the virus antigen (AG) vial thiols in cysteine moieties

For initial testing, MNP coated with APTES ((3-aminopropyl)triethoxysilane, Carl Roth, Germany) were used as an exemplary particle system. These MNPs are coated with APTES and produced by alkaline precipitation as described in (Friedrich et al. 2022b). The MNPs coated with APTES have a typical hydrodynamic diameter of about 170 to 200 nm as described in recent works (Friedrich et al. 2022b; Vogel et al. 2022). The SARS-Cov-2-S1 proteins (SARS-Cov-2 (2019-nCoV), MERS-Cov-S1, and the SARS-Cov-2-N (Sino Biological, China) were covalently attached to the surface of the particles by reacting SBA (N-succinimidyl bromoacetate) with the amino groups of APTES before linking the cysteines or lysines present in the protein via the bromoacetyl residue (Fig. 1). A 0.05 M borate buffer at a pH of 8.5 was used for the functionalization steps. The iron concentration during processing was adjusted to 1 mg Fe/ml together with an amount of 20 mM SBA dissolved in DMF (Carl Roth, Germany). The functionalization was carried out on a shaker for 2 h at 1400 rpm at room temperature. After that, the particles were washed several times with buffer solution. The obtained particles were redispersed in borate buffer for binding of SARS-Cov-2-S1, MERS-Cov-S1, and the SARS-Cov-2-N protein with a concentration of 10 μg protein per 100 μg Fe followed by another incubation of 2 h at 1400 rpm. The resulting functionalized particles were then magnetically washed several times with doubly distilled H_2_O. Following the separation of the magnetic particles from supernatants after synthesis and washing steps, they were analyzed by UV measurements at 280 nm to ensure successful binding using the SpectraMax iD3 Plate reader (Molecular Devices, San José, USA). Iron concentration as well as hydrodynamic size were acquired as recently described (Friedrich et al. 2022b; Kappes et al. 2022).

### Characterization of functionalized MNP

Concerning their physicochemical features, MNPs were analyzing regarding their size, iron content according to previous studies (Friedrich et al. 2021; Mühlberger et al. 2019; Stein et al. 2020). Their iron content in milligram per milliliter was investigated after diluting them 1:20 in deionized H_2_O, liquefying them in 65% nitric acid with atomic emission spectroscopy (AES), using Agilent 4200 MP-AES with an iron solution of 1000 mg Fe/l as an external standard (Bernd Kraft, Duisburg, Germany). MNP particles were diluted to the desired concentration with deionized water for all experiments. Transmission electron microscopy (TEM) images were acquired using the CM30 TEM/STEM (Philips, The Netherlands) operating at 300 kV acquired with a CCD camera, a Tietz Fast Scan-F114 (Tietz Video and Image Processing Systems GmbH, Gauting, Germany). Dispersions at a concentration of 100 µg Fe/ml were dropped onto a carbon-coated copper grids (Plano, Germany) an air-dried at RT.

### Detection of particle-bound antibodies using flow cytometry

To test the binding selectivity to the correct antibody, flow cytometry was performed using a Gallios flow cytometer (Beckman Coulter, Fullerton, CA, USA). First, the antibodies for loading control of MNP-S1 on the MNP, SARS-Cov-2-Spike antibody was diluted in PBS with 0.1% BSA to a concentration of 10 μg/ml (for loading control only). Furthermore, 10 μg/ml of the respective individual antigens namely SARS-Cov-2-Spike, SARS-Cov-2 nucleocapsid, and MERS-Cov-Spike (Sino Biological, China) was loaded on the MNP. For the measurement, 25 μl of MNP-antigen dispersions (100 μg Fe/ml) was added in a 0.5-ml Eppendorf cap. Subsequently, 25 μl of antibody dilution (1:20,000) or only buffer as a control was added. Samples were incubated for 1 h at 4 °C. For washing, samples were centrifuged at 18,000 g for 10 min, the supernatant discarded, and the MNPs redispersed in buffer. For detection, particles were further incubated with fluoresceinisothiocyanat (FITC) labeled protein A (10 μg/ml) for 1 h at room temperature (RT), which binds specifically to the Fc-region of antibodies. Finally, MNP-antigen was washed as described before, redispersed and diluted 1:25 in buffer, and analyzed for their fluorescence by using FACS. The fluorescence bleeding through was eliminated by electronic compensation. The acquired data were analyzed with Kaluza software version 2.1 (Gallios, Brea, USA).

### Detection of antibodies in camel sera using ELISA

The strength of the immune response was determined using the cPassTM SARS-CoV-2 Neutralization Antibody Detection Kit, GenScript, China, and anti-MERS-CoV ELISA (IgG), Euroimmune, Germany. The test was performed following the instructions in the kit. The commercial Anti-MERS-CoV ELISA Camel (IgG) kit is based on the MERS-CoV S1 subunit as described before (Drosten et al. 2014). Diluted serum samples (1:101 in sample buffer) were first incubated at 37 °C for 30 min in microplate wells coated with recombinant structural MERS-CoV S1 protein. The secondary antibody, an enzyme-labeled anti-camel IgG, was then added to the wells in a second incubation step (37 °C, 30 min). The substrate was then added to the wells for 15 min at room temperature developing into a color reaction. Washing steps were performed in between, according to the kit instructions. The extinction value was measured at OD 450-nm light and semiquantitative evaluations were performed by using the ratio values (sample value extinction over calibrator value extinction). As recommended by EUROIMMUN, a ratio < 0.8 was set as a negative result and a ratio ≥ 1.1 as positive. The cPass™ SARS-CoV-2 Neutralization Antibody Detection Kit is a blocking ELISA that detects all types of neutralizing antibodies against SARS-CoV-2. Diluted serum samples (1:9) and the controls in sample dilution buffer were mixed with the diluted HRP-RBD solution with a volume ratio of 1:1. This mixture was incubated at 37 °C, for 30 min, and then added to the microplate wells coated with purified receptor binding domain (RBD) protein from the viral spike (S) protein. Followed by the incubation step (37 °C, 30 min), washing steps were performed according to the kit instructions. The substrate was then added to the wells for 15 min at room temperature leading to a color reaction and added stop solution to quench the reaction. The absorbance in the microtiter plate was read using a plate reader at 450 nm. The inhibition percentage was calculated according to the kit instruction. Samples with percentage inhibition values lower than 30% were considered negative, and samples with percentage inhibition value greater than or equal to 30% were considered positive.

### Detection of particles bound antibodies using COMPASS

All samples were prepared according to established procedure by pipetting 25-μl camel sera samples derived from 5-ml stock sera (sera samples were diluted 1:200 in buffer for the measurements) to 25 μl functionalized MNP suspension with a concentration of 100 μg Fe/ml. After an incubation time of 1 min, measurements were performed 5 times without averaging. The sequence of samples was reference sample containing buffer (PBS with 1% BSA) and selected camel sera (negative sera, Sera from SARS-CoV-2-S1, SARS-CoV-2 whole virus, and MERS-RBD immunization). Individual measurements were repeated 3 times resulting in total 15 measurements. The acquisition time for each experiment was 10 ms with a minimum repetition time of 1 s. Due to increasing the hydrodynamic diameter of the functionalized MNPs, the mobility (effective viscosity) of the particles change. These small changes can be measured with the critical offset magnetic field approach resulting in phase changes on selected critical points. The selection of the critical point, here the first critical point of the fifth higher harmonic, is done in a prior calibration step setting the required AC/DC ratio (Vogel et al. 2022). The excitation frequency is 20 kHz and the respective AC field had a strength of 15 mT. The simultaneously applied permanent magnetic field (DC) had a strength of 10 mT. The values for AC are exactly adjustable while the values for DC are adjusted to the respective harmonics (automatic calibration).

### Statistical analysis

Statistical analyses were performed using GraphPad Prism 9.0 (GraphPad Software Inc., San Diego, CA, USA). One-way ANOVA with post hoc Turkey’s test and/or Kruskal–Wallis test with Dunn’s post hoc test was employed to determine statistical significance and *p* values < 0.05 were considered significant.

## Results

### Protein modified magnetic nanoparticles for antibody detection

**Fig. 2:**
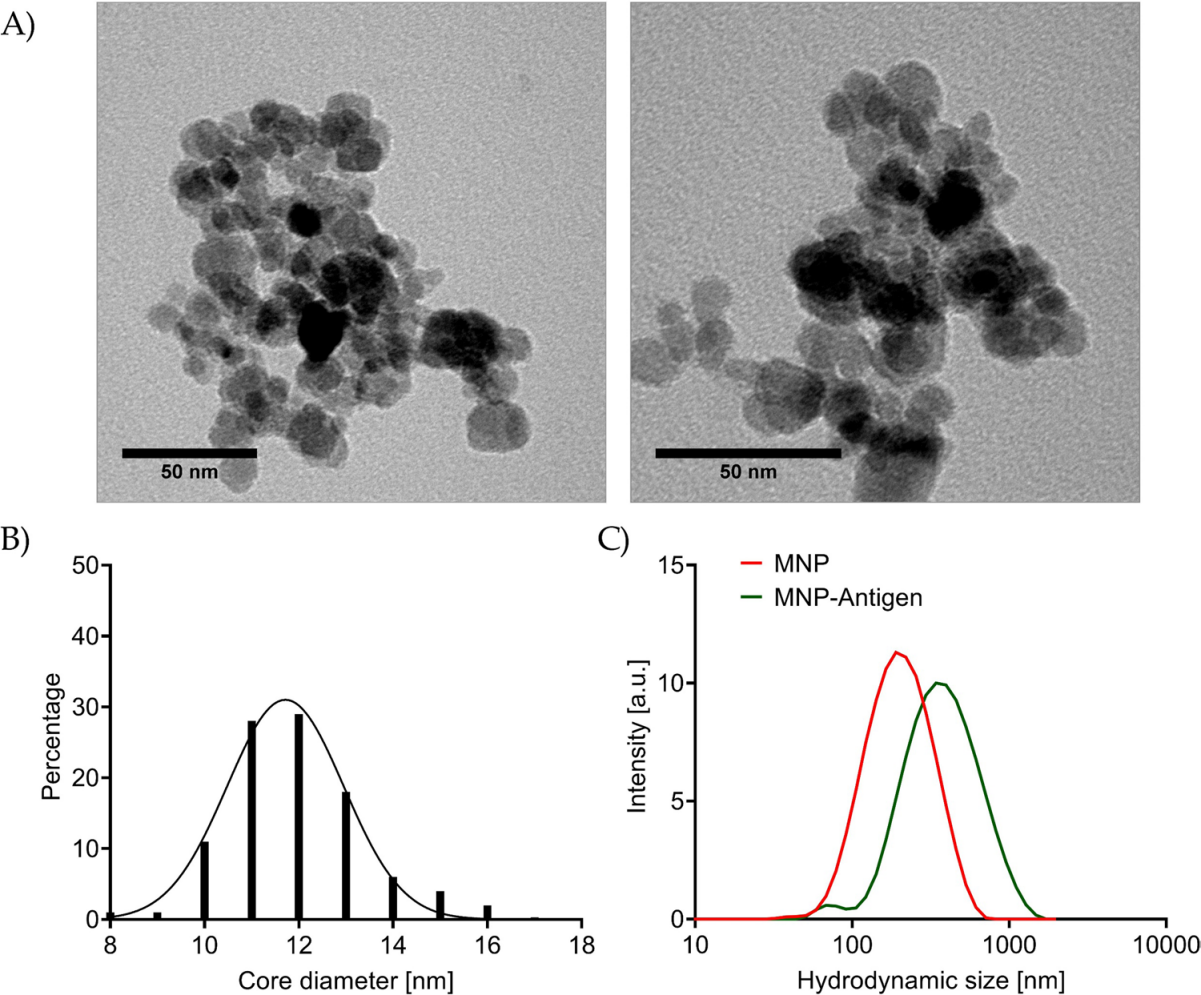
**A** Bright field TEM-images of MNP. **B** Core size distribution of MNP. **C** Hydrodynamic size of MNP and MNP-antigen (SARS-Cov-2-S1)

MNP as used in this study are a very versatile tool that can easily be modified to detect antibodies, antigens, or cells by attaching selected functional bait molecules as in our case viral antigen on the surface of the particles. MNP as seen in a TEM image (Fig. 2) present such a versatile particles system as in this study via the -NH_2_ groups of silanized particle surface.

**Fig. 3:**
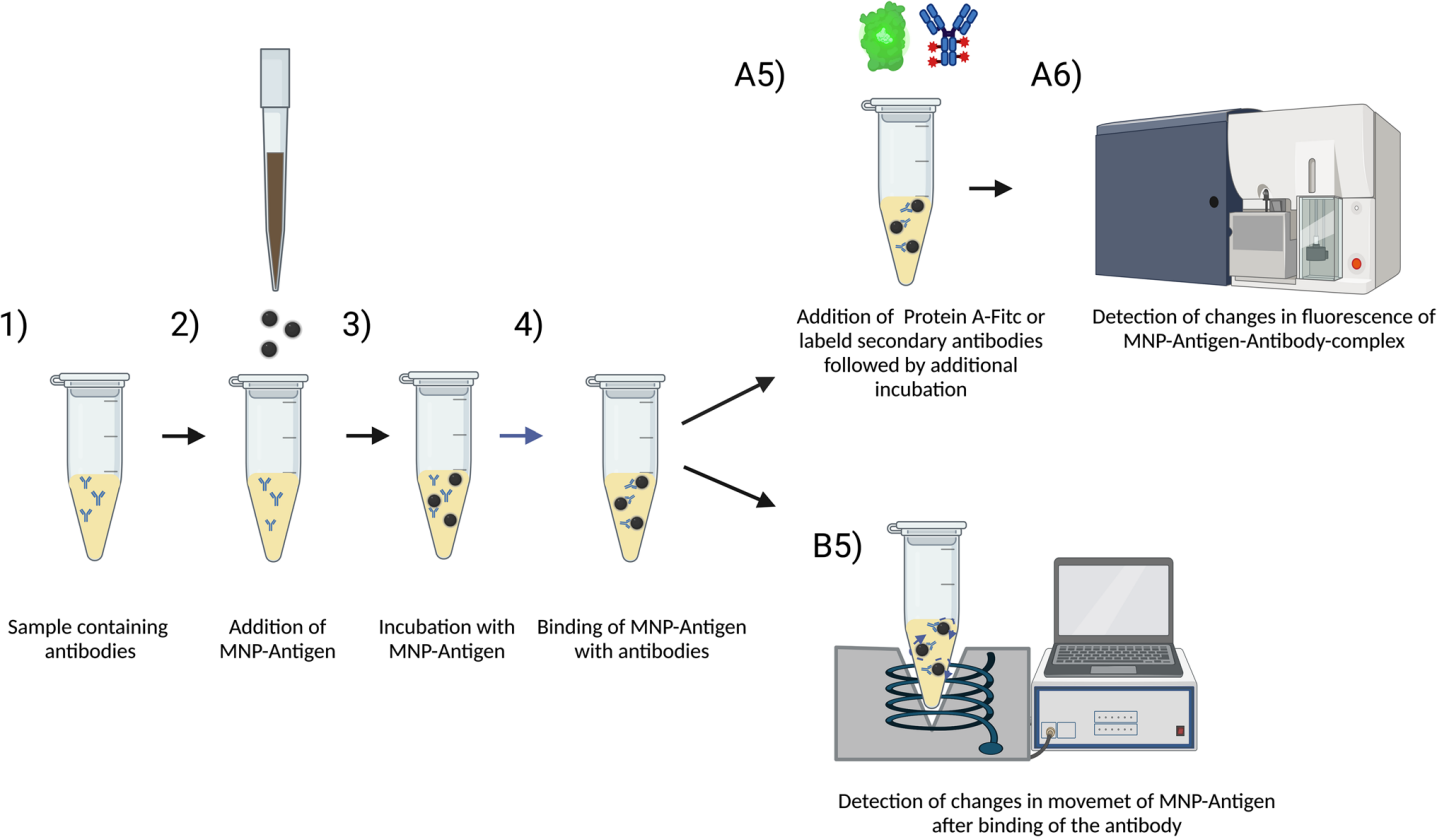
Scheme of MNP-antigen-based detection using FACS or COMPASS analysis. To samples that contain antibodies (1) the matching MNP-antigen are added (2). After incubation (3–4), the samples can be directly measured using COMPASS (B5) or have to undergo a second incubation step with fluorescent antibodies (A5) before measuring using flow cytometry. Created with BioRender.com

In this study, we used different viral proteins by exchanging the bound bait protein on the MNP. After binding and washing, the hydrodynamic size of the particles was about 330 nm and the particles were stored in water. However, it should be pointed out that the core size for this multicore particle remains almost unchanged after functionalization as shown in other studies using similar techniques, showing a mean diameter of about 12 nm (Friedrich et al. 2022a, b). Figure 3 shows the approach of the antibody detection in this study. It should be stated that the detection using FACS needs an additional incubation step with corresponding detection antibodies or in our case protein A-FITC to detect the binding of the antibodies on the particles.

### Predevaluation of antibody binding ability using FACS

**Fig. 4:**
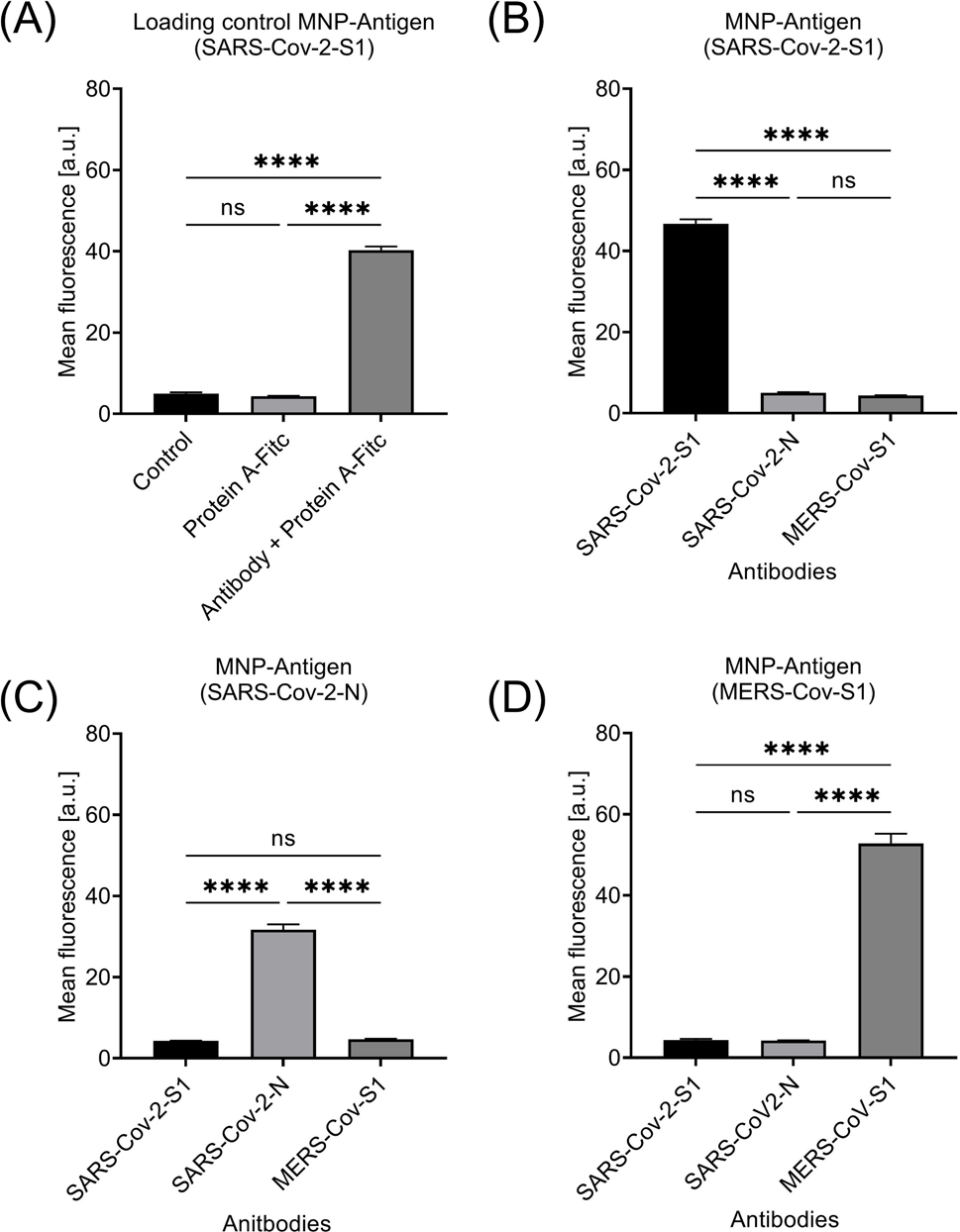
Detection of antibody binding on protein functionalized MNP using flow cytometry. **A** Loading control showing no strong autofluorescence or unspecific binding of protein A-FITC to the MNP. **B**–**D** Detection of antibodies by different antigen functionalized MNPs using flow cytometry. Shown are the mean values of three independent experiments. One-way ANOVA with post hoc Turkey’s test was used to determine the significance with ****p < 0.0001. *p* values above 0.05 were considered non-significant (ns)

A very common approach is to measure and quantify the amount of different substances like aptamers, antibodies, or antigens to particle-based systems which is the measurement of the fluorescence of the individual particles by flow cytometry (Helmberg et al. 1997; Neves et al. 2021; Yang et al. 2003). However, such techniques require specific detection antibodies or labeled protein A. Such are often very expensive and also in case of camels cannot detect the whole magnitude of antibodies in the sample as protein A cannot bind to nanobodies that are a typical antibody found in camels. Figure 4 displays the different functionalized particles in combination with all tested antibodies. It was found that only if the complementary combination of antigen and antibody was present, an increase in the fluorescence signal was present (Fig. 3 A). Interestingly, the SARS-CoV2-N protein (Fig. 4 C) shows a weaker fluorescence increase in comparison to the spike antibodies of SARS-CoV2-S1 (Fig. 4 B) and MERS-Cov-S1 (Fig. 4 D).

### Detection of different antibodies in camel sera using COMPASS

Compared to currently used diagnostic in vitro techniques, the use of the new COMPASS technique offers an adaptable, faster, and even more reliable method to detect antibodies (Vogel et al. 2022) and thereby improve the screening of viral diseases in camels. In a first trial, this study showed that the antibodies could be detected without prior additional preparation in very short times even at very low concentrations. To evaluate the efficiency of such a device and test the specific binding of the antibodies on the MNP, three different immunized camel sera samples were screened with different functionalized MNPs. All camel sera have also been analyzed with standard ELISAs that confirmed the results of the magnetic measurements.

**Fig. 5:**
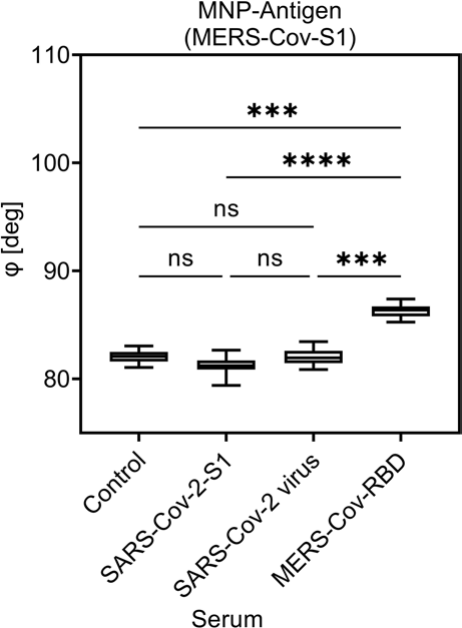
Detection of viral antibodies using COMPASS and MNP-MERS-Cov in different camel sera. Shown are the mean value of the phase of higher harmonic (*n* = 5th) for three individual experiments. Each sample was measured five times. The Kruskal–Wallis test with Dunn’s post hoc test was used to determine the significance with *p* ≤ 0.0002; ****p < 0.0001. *p* values above 0.05 were considered non-significant (ns)

**Fig. 6:**
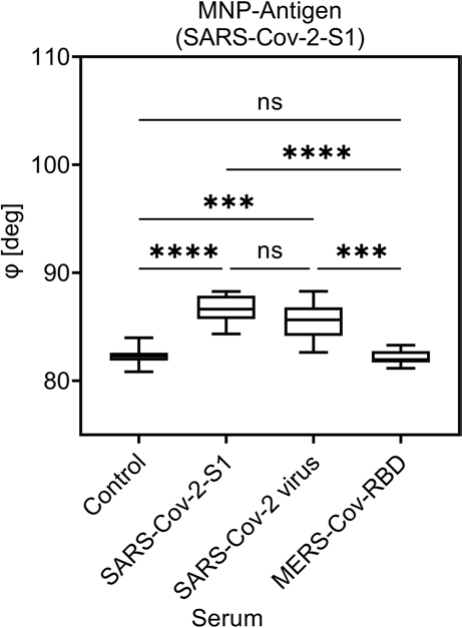
Detection of viral antibodies using COMPASS and MNP-SARS-CoV-2-S1 in different camel sera. Shown are the mean value of the phase of higher harmonic (*n* = 5th) for three individual experiments. Each sample was measured five times. The Kruskal–Wallis test with Dunn’s post hoc test was used to determine the significance with ***p ≤ 0.0002; ****p < 0.0001. *p* values above 0.05 were considered non-significant (ns)

**Fig. 7:**
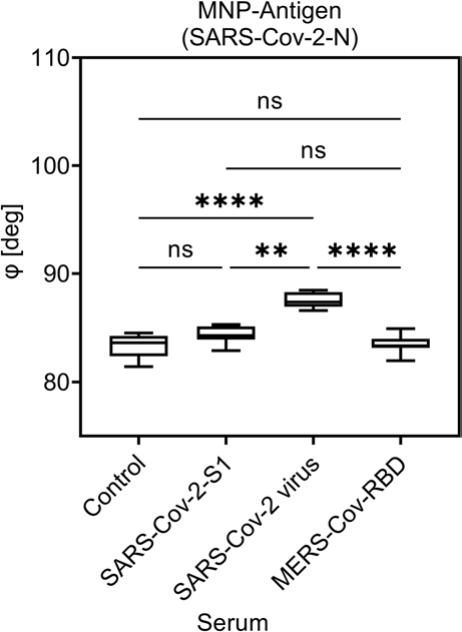
Detection of viral antibodies using COMPASS and MNP-SARS-Cov-2-N in different camel sera. Shown are the mean value of the phase of higher harmonic (*n* = 5th) for three individual experiments. Each sample was measured five times. The Kruskal–Wallis test with Dunn’s post hoc test was used to determine the significance with ** *p* ≤ 0.01; ***p ≤ 0.0002; ****p < 0.0001. *p* values above 0.05 were considered non-significant (ns)

The graphs in Figs. 5, 6, and 7 show the phase *φ*_n_ on selected critical point of the fifth higher harmonic (*n* = 5th) against the control sample (negative sera). A significant signal in phase was observed for the specific sera but not for the control or the non-specific sera.

Also, the variance of positive samples was high they still showed a significant difference compared to negative (control or other non-binding antibody) samples (Fig. 6). The used system is able to measure a sample multiple times within seconds to ensure a clear value. For visualization, negative camel sera (control) can also be used as reference and therefore be normalized by the COMPASS device to allow a fast screening of camel populations by simply detecting higher phase values than controls. As shown in Fig. 7, a clear distinction between camels that were immunized with the whole SARS-CoV-2 virus and those that only were immunized with the S1 protein was possible by the use of the correct functionalized MNP.

## Discussion

Screening of diseases in livestock is a very important aspect to avoid severe economic losses in livestock farming and to mitigate the hazard potential of human pathogenic zoonoses (Filippitzi et al. 2017). In the breeding of camels, infections with coronaviruses play a role among other diseases. To identify the species within this virus family in a differentiated way and to use simple but at the same time robust methods is a challenging task. In current practice, a variety of different methods is used for this purpose (Glais and Jacquot 2015; Simmonds and Aiewsakun 2018).

In our study, we deployed magnetic particles functionalized with certain antigens in order to immobilize the complementary antibody which was demonstrated via flow cytometry as an independent reference method. In the following, we took advantage of certain material properties of functionalized magnetic particles.

A clear correlation between antigen functionalization and binding to the corresponding antibody could already be confirmed in the measurements with flow cytometry.

Interestingly, the results in Fig. 4 show a difference in the mean fluorescence signal when comparing the values for SARS-Cov-2 N functionalized MNP compared to MNP functionalized with S1 proteins of SARS-Cov-2 or MERS-Cov. This could be attributed to a lower affinity of the nucleocapsid (N antigen) to be bound to the particles as the linkage is established via the -NH_2_ with a lower affinity and not the thiol groups, which are not present in this protein. In conclusion, due to the required washing steps and preparation of the samples for FACS measurements, it can be assumed that partially N antigen is released from the MNP.

However, there was still a visible and significant increase in fluorescence for these particles after applying the correct antibody. In a later stage of translation into veterinary application, problems like this could be solved by adding suitable terminal linking sequences or tags to the used recombinant bait protein.

For the results of the COMPASS experiments shown in Figs. 5, 6, and 7, the phase in the vicinity of the 1st critical point of the 5th higher harmonic is used. It has to be emphasized, that due to the magnetic gradient generated by a permanent magnet, a broad range of offset magnetic fields allows the simultaneous acquisition from multiple critical points from different harmonics besides the used one (Vogel et al. 2022).

Our method competes to established molecular biological methods. ELISAs are widely used for the detection of disease-specific antibodies. Antigens are presented on solid phases and incubated with the analyte solution to detect the target. Extensive purification steps are required and the signal is ultimately based on a color reaction, which can be susceptible to various interferences (Bommana et al. 2019). Gold-coated nanoparticles are a special type of ELISA, which, due to the solid-phase geometry and the specific binding properties of gold to thiols, lead to faster binding kinetics and shorter times to equilibrium (Zhang and Meyerhoff 2006). The readout is performed here using a conventional sandwich ELISA concept. However, a direct detection from the diluted blood serum is hardly possible. To overcome the difficulties of extinction-based colorimetric assays, flow cytometry can be used. Here, beads are functionalized with a bait molecule to capture the analytical target (Cáceres-Martell et al. 2021). Flow cytometry analyses are very powerful and the equipment infrastructure is available in many research and routine laboratories where laboratory personnel are also appropriately trained in this methodology. There are also useful assay kits and various possibilities to generate customized methods on the basis of commercially available functionalizable beads (Lim and Zhang 2007). Bead-based immunoassays for use in flow cytometry therefore have several advantages. Nevertheless, washing steps have to be performed between the individual process stages and the measurement is also time and resource consuming. Hence, the detection of antibodies by flow cytometry is similar time-consuming as regular ELISA methods since it also requires washing and long incubation steps and is dependent on the speed of the deployed flow cytometer device (Neves et al. 2021).

A completely different approach to the detection of pathogens is the detection of their RNA or DNA fragments. Minimal traces of the respective genetic material are detected very sensitively. PCR-based methods are well established, but answer a completely different question, namely the presence of the pathogen, regardless of whether it has led to an infection or not (Eldin et al. 2019). The high sensitivity is at the same time the biggest system-related disadvantage of this method and PCR is particularly susceptible to contamination. Especially in laboratories that frequently test the same analytical targets, false-positive results often occur (Huggett et al. 2020).

All these methods have in common that they cannot be performed on site and require complex and time-consuming sample logistics. To circumvent such difficulties, POC methods are often used, which are easily portable and readily available. However, the quality is variable and the analysis, especially at low analyte concentrations, is often poor in terms of analytical performance (Corman et al. 2021).

The present work demonstrates that by the use of COMPASS, we were able to detect antibodies highly sensitive in camel sera. In general, it can be stated that the presented method can efficiently identify the presence of antibodies in standard samples of camel sera. According to previous studies this can also be used for quantification (Vogel et al. 2022), there a limit of detection of about 7 pM was determined. Nevertheless, this method offers a fast identification of specific antibody presence in samples.

Moreover, we were able to discriminate different antibodies against corona virus subgenera namely SARS-CoV-2 and MERS. The MPS-based system reduces the screening time drastically compared to standard ELISA tests as the sera can be applied directly and the results are available almost instantly. Furthermore, it is possible to make statements about the choice of particle-bound antigens as to whether the initiated antibody response is based on vaccination with a specific antigen—mRNA or protein based—or on infection/vaccination with the complete virus. The COMPASS method can easily be modified by exchanging the bait molecules on the MNP. The method does not require additional washing steps during the process compared to standard ELISA and flow cytometric methods as the antibody directly binds to the MNP resulting in a very sensitively measurable change of movement.

In conclusion, still further studies and assay developments are required to obtain an easy to use assay method to allow an exact quantification of the antibody amount to meet standards in conformity with approvals. We believe that this method can further be used in the prevention of newly emerging zoonotic viral diseases as well as diseases that already are prevalent by fast and portable use of this technique. The feasibility of this method essentially depends on addressing further relevant analytical targets, providing the corresponding SPIONs with the appropriate complementary binding capture ligands, and proving the application in practice. Intensive work is currently underway to further expand the range of applications. The first results shown here regarding antibody detection against SARS-Cov 2 and MERS-Cov are a promising start. This shows the diagnostic potential due to the advantages of the method such as little time to result, high sensitivity, and selectivity with minimal technical effort and portability of the device.

## Data Availability

The data that support the findings of this study are available from the corresponding author upon reasonable request.
